# Trumpet sounds emitted by male sperm whales in the Mediterranean Sea

**DOI:** 10.1038/s41598-021-84126-8

**Published:** 2021-03-12

**Authors:** D. S. Pace, C. Lanfredi, S. Airoldi, G. Giacomini, M. Silvestri, G. Pavan, D. Ardizzone

**Affiliations:** 1 Department of Environmental Biology, Sapienza University of Rome, Rome, Italy; 2 Tethys Research Institute, Milan, Italy; 3 Department of Earth and Environmental Sciences, CIBRA, University of Pavia, Pavia, Italy

**Keywords:** Zoology, Animal behaviour

## Abstract

Sperm whale trumpets are sounds only occasionally documented, with a well recognisable and stereotyped acoustic arrangement. This study investigated the acoustic features of the trumpets and the context in which these sounds were recorded, using acoustic data collected over 22 years, in the Pelagos Sanctuary area (North-Western Mediterranean Sea). Analysed trumpets (n = 230), recorded at the beginning of a dive after the whale fluke-up, comprised a series of acoustic units organized in short sequences. Acoustic parameters were derived for the entire trumpet and for each distinguishable unit in a trumpet. Overall, trumpet durations and their initial frequencies were higher in recordings collected when multiple whales were visually or acoustically detected in the observation area. The identity of 68 whales was assessed through photo-identification, with 29 individuals producing trumpets within and between years. The variability of the acoustic parameters appeared to be higher within the same individuals rather than between different individuals, suggesting an individual plasticity in composing and arranging units in a trumpet. Different click patterns were observed before and after the trumpets, with more complex sequences when (1) other whales were visually/acoustically detected, and (2) individuals were in suitable foraging sites (i.e., canyon areas). Trumpets were commonly followed or preceded by click patterns suited for communication, such as codas and/or slow clicks. Significant relations between the trumpet emission and the male-only long-range communication click pattern (i.e. slow clicks) emerged, supporting the hypothesis that a trumpet is a sound emitted by maturing/mature males in feeding grounds. This study provides the first evidence that trumpets were conserved in the sperm whale acoustic repertoire at the decadal timescale, persisting across years and individuals in the same area. This persistence may be functionally specific to foraging activities performed by males in a well-established feeding area.

## Introduction

Marine mammals rely heavily on sounds as their primary means of communication and sensing their word; where acoustic cues serve a fundamental role in all exchanges between individuals, from social interactions to the coordination of group activities^1–3^. Some of these sounds have been investigated quite extensively in several species such as sperm whale (*Physeter macrocephalus*), and their significance and diversity are relatively well-established. Sperm whales mostly produce a number of sharp onset, broadband, evenly spaced pulses of decaying amplitude known as ‘clicks’, with different properties and repetition rates, and a bandwidth of 100 Hz–30 kHz^4–8^. Clicks—generated by the massive sperm whale nasal complex—may be temporally arranged in different patterns, having both echolocation and communication functionality^4,7,8^. Usual clicks and creaks^9,10^ are produced at depth and appear to be used primarily in searching for food and targeting the prey, respectively^8^. Codas, generally emitted at the surface, are stereotyped patterns of clicks thought to serve in social communication in both sexes^8,11^. Slow clicks, which are heard in the presence of mature or maturing males^5,7,12^ at depth and at the surface, seem to be related with the sperm whale mating system, as long-range communication for attracting females or in male-male competition^8^. Long-range communication between males in foraging grounds has been also reported, suggesting that slow clicks functionality may vary depending on the behavioural context^12^. Some additional defined click patterns of surface creaks^8^ (i.e. coda-creaks^10^), rapid/fast clicks, and chirrups^9,13^) have also been described in the acoustic repertoire of the species, and are possibly used for scanning their social partners^8^.

Sperm whales are also able to produce non-click sounds^8^. These include “squeals”, with a possible communicative social function^13,14^, ‘pips’^13^, “short trumpets”^13^ and “trumpets”^9^.

Little information is available in the literature regarding trumpets (Table 1). Gordon^9^ wrote the earliest reference of these narrow-band sounds with harmonics and described these calls as similar to trumpets sounds produced by elephants: “This sound, like a muffled trumpeting call of an elephant, was recorded very clearly on three occasions after the fluking-up of one particular whale and before it started clicking”. Then, several research groups have recorded and identified occasional trumpet sounds^10,13,15–19^. These studies showed that sperm whale trumpets appear as tonal sounds relative to human hearing and in their spectrographic representation, consisting of units lasting about 0.2 s each and arranged in short sequences, with energy up to 20 kHz. It has been reported that the number of units ranges from 2 to 18 (Table 1), and the entire sequence in a trumpet takes between 0.6 and 4.3 s^18,19^. Even trumpets seem like tonal sounds, their structure can be seen as a fast sequence of evenly spaced pulses, but with varying inter-pulse intervals. The waveform and the harmonic structure support the hypothesis of the pulsed nature of trumpets^20^ and suggest their possible source in the sperm whale monkey lips (i.e., specific valves for sound generation located in the nasal complex and associated with small fat bodies, the dorsal bursae, which can vibrate in the air current and produce sound waves in adjacent tissues^21–23^).

**Table 1:** Synoptic table of the studies on the sperm whale trumpets world-wide.

N. of analyzed/reported trumpets	Time period	N. of units per trumpet	Trumpet total average duration (s)	Trumpet initial frequency (Hz)	Trumpet final frequency (Hz)	Average unit duration (s)	Time interval between the end of a unit and onset of next unit (s)	Unit initial frequency (Hz)	Unit final frequency (Hz)	Time interval form the trumpet to the first usual click (s)	Location	Ref	Notes
3/3	1981–1984	–	–	–	–	–	–	–	–	–	Sri Lanka (Indian Ocean)	^9^	
?	1985–1989	–	–	–	–	–	–	–	–	–	Galapagos Islands (Pacific Ocean)	^10^	
83/83	1998	–	–	–	–	–	–	100–1000	–	–	North Sea/Atlantic Ocean	^13^	Termed as ‘short trumpets’ to distinguish them from Gordon's (1987) observations
0/3	1996	–	–	–	–	–	–	–	–	–	NW Mediterranean Sea	^15^	
2/2	2001	–	–	–	–	–	–	–	–	–	NW Mediterranean Sea	^16^	
11/93	1995 2000–2003	2–15	2.3 (0.6–3.5)	–	–	0.2	0.1	500	–	28 (8–102)^a^ n = 28	Mediterranean Sea (n = 9) Norway (n = 1) Gulf of Mexico (n = 1)	^17^	
0/45	2001–2003	–	–	–	–	–	–	–	–	–	NW Mediterranean Sea	^18^	On 19 occasions trumpets were followed by a short sequence of clicks (usually 2) with an ICI of about 5 s
29/29	2006–2016	5–18	2.3 (1.12–4.35)	438.5 (260–859)	690.5 (510–1001)	0.21 (0.04–0.24)	0.06 (0.9–0.34)	352 (247–494)	579 (420–892)	21 (3–183)	Tyrrhenia Sea (Italy)	^19^	

Results by Teloni and colleagues^18^ also showed that trumpets are produced by the same individual at the start of the descendant phase of a dive (at shallow depth) before the onset of a usual click sequence (confirming the observation reported by Gordon), and that the time interval from the trumpet to the first usual click averaged 28 s. Teloni^17^ reported that in some instances the trumpet is preceded by codas, explaining this as a sort of preparation of the phonation organ for the following click emissions with echolocation function.

Trumpets are actually supposed to be by-products of the click generation mechanism when the sperm whale nasal complex is adjusted to switch from a configuration appropriate to respiration to one suitable for echolocation clicks^9,18^. Another suggestion is that the trumpet could be produced by a threatened whale as an alarm call due to the presence of the vessels^9,13^, but the variability, the stability and the functional significance of these sounds remains uncertain. The modest source level and the apparent lack of directionality^18^ seem to exclude echolocation, and the possible trumpets’ communicative role as a signal (i.e., selected for conveying information to recipients to elicit responses that result in fitness consequences^24,25^) or a cue (i.e., not shaped by natural selection for the purpose of transmitting information, but able to provide information to others as a by-product of an activity^24,25^) is not clearly inferred from existing data.

Here, we present the features of sperm whale trumpets recorded in the Pelagos Sanctuary area (Mediterranean Sea), with the aim of expanding the knowledge on these less studied sounds and offer new insights on the emission context.

## Materials and methods

### Study area

**Figure 1:**
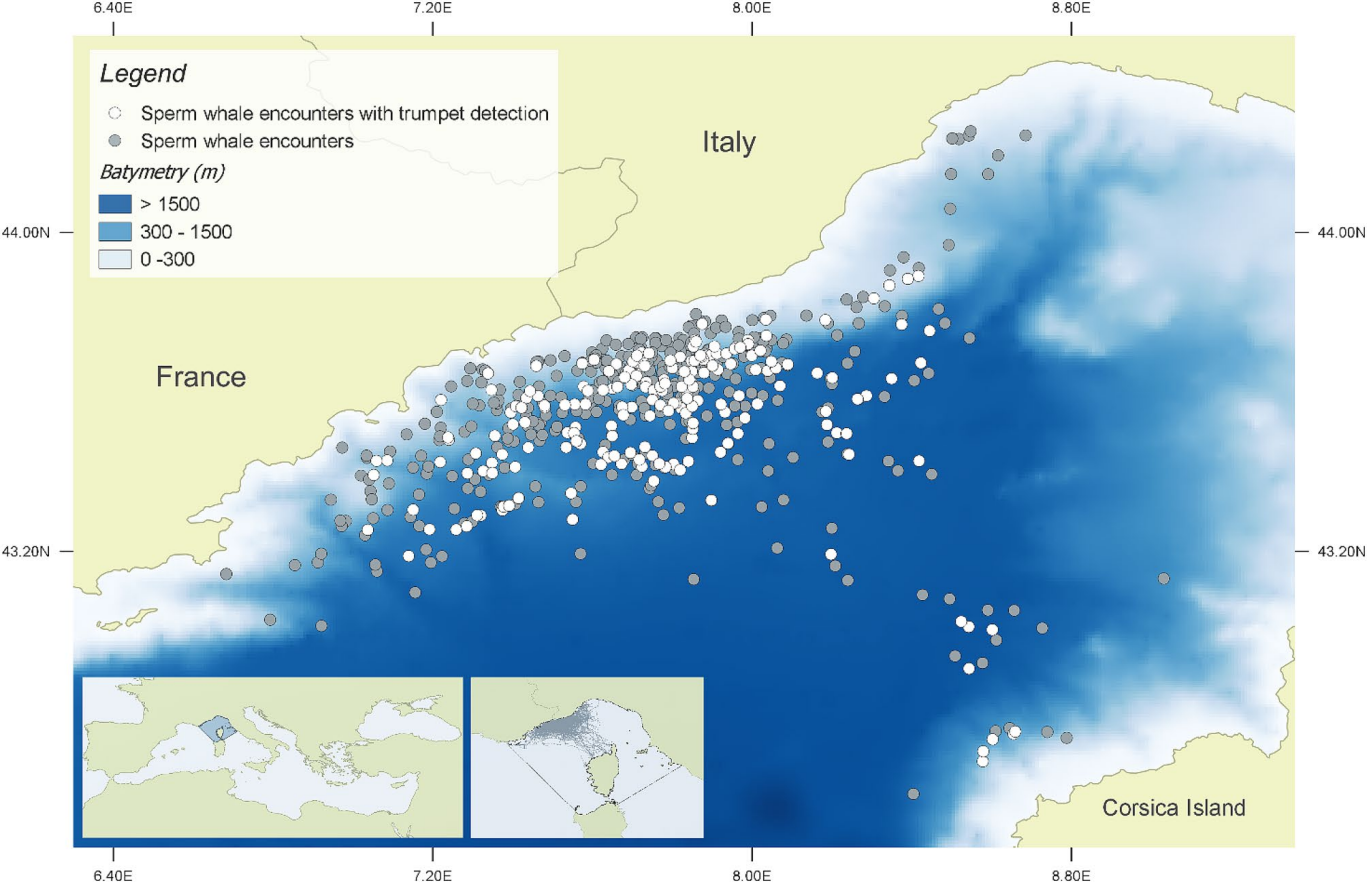
Tethys Research Institute/CSR project study area. All sperm whale encounters between 2007 and 2018 are shown. White dots indicate location of trumpets recordings. TRI/CSR cruise track lines, and the Pelagos Sanctuary borders are shown in the panel. Map created using the Free and Open Source QGIS.

The study site is located in the north-western portion of the “Pelagos Sanctuary for Mediterranean Marine Mammals”^26^ (Fig. 1). The area is characterized by a complex geomorphology with a narrow continental shelf, deeply incised by several submarine canyons, followed by offshore waters deeper than 2500 m. The presence of a permanent frontal system and the interaction between geomorphologic and oceanographic factors makes the region one of the most productive of the Mediterranean^27,28^.

Sperm whales have been reported in the area during the summer period since 1990^29,30^, with predominant foraging activities^31,32^. The estimated length of the encountered individuals suggests the area is primarily used by males^33–36^ generally swimming or diving alone, or seen alone at the surface^37^, while females and calves in social units (sensu Whitehead^8^) are infrequently sighted^38^ or stranded^39^. Sperm whale habitat preference is related to regions with well-defined depth and slope gradients, as in other Mediterranean locations^30,31,40–44^. Sperm whale occurrence in this study area has been reported over a 25-year period^30^, providing key information on the population status of a species in suspected decline in the Mediterranean Sea^45^ and listed as Endangered in the IUCN Red List.

### Data collection and field procedures

We analysed two different sperm whale acoustic datasets. The first one derives from the Cetaceans Sanctuary Research long-term research program (1990-ongoing) run by Tethys Research Institute (TRI), Italy, and includes sperm whale recordings collected between 2007 and 2018. The second one originates from an acoustic campaign conducted by CIBRA-University of Pavia, Italy, in 1996. We used this CIBRA historical dataset as it contains the first trumpet recordings in the Mediterranean Sea and accounts for the permanence of these sounds in the basin.

TRI recordings were collected during visual and acoustic surveys conducted in spring/summer (May–September) using sailing vessels of 15–21 m. Two observers, positioned one at each side of the vessel at a height of approximately 3 m above the sea surface, visually scanned for cetaceans by using 7 × 50 binoculars during daylight. Visual effort was performed under ‘favourable conditions’ only (i.e. the vessel speed averaged 5–11 km h^−1^ in sea state conditions corresponding to a Beaufort scale lower than 3). Acoustic surveys were also conducted in higher sea state conditions.

A dedicated laptop, connected to a GPS receiver, automatically acquired and logged the GPS track every minute. The International Fund for Animal Welfare (IFAW) software Logger 2000 and Logger 2010, and the software PAMGuard (version 1.15) implemented by the University of St. Andrews were used for data logging. Acoustic detections were performed using a stereo hydrophone array incorporating two hydrophones (BENTHOS AQ4—frequency range 10 Hz to 15 kHz − 3 dB) with 2 pre-amps (Magrec HP02 with high pass filters set to − 3 dB at 100 Hz) towed on a 200 m cable. The system was connected to the laptop through an audio interface (Sample rates: 44.1 and 96 kHz, 16-bit resolution). Rainbow Click IFAW software (http://www.marineconservationresearch.co.uk/downloads/logger-2000-rainbowclick-software-downloads/) or the “Click Detector” PAMGuard module (https://www.pamguard.org/devDocs/clickDetector/package-summary.html) were used to detect and track the sperm whale clicks. Once the sperm whales were detected, the vessel was maneuverer to determine the bearing of the vocalizing focal animal relative to it. In case of more than one clicking sperm whale, the focal animal was labelled as the one producing the more intense sound. To track the focal animal, the stereo signal was analysed using time of arrival differences between the same clicks on the two channels to estimate the bearing of each click source^46,47^. This approach allowed to track the sperm whale until the end of its dive (i.e., the time the whale was first sighted at the surface^48^) having as final goal the identification of the animal through photo-identification. When the tracked whale stopped clicking, the acoustic operator informed the visual observers, since cessation of clicking was usually an indication of the end of the dive. When the sperm whale was sighted at the surface, surfacing time, geographic position and respiration pattern, were also collected.

**Figure 2:**
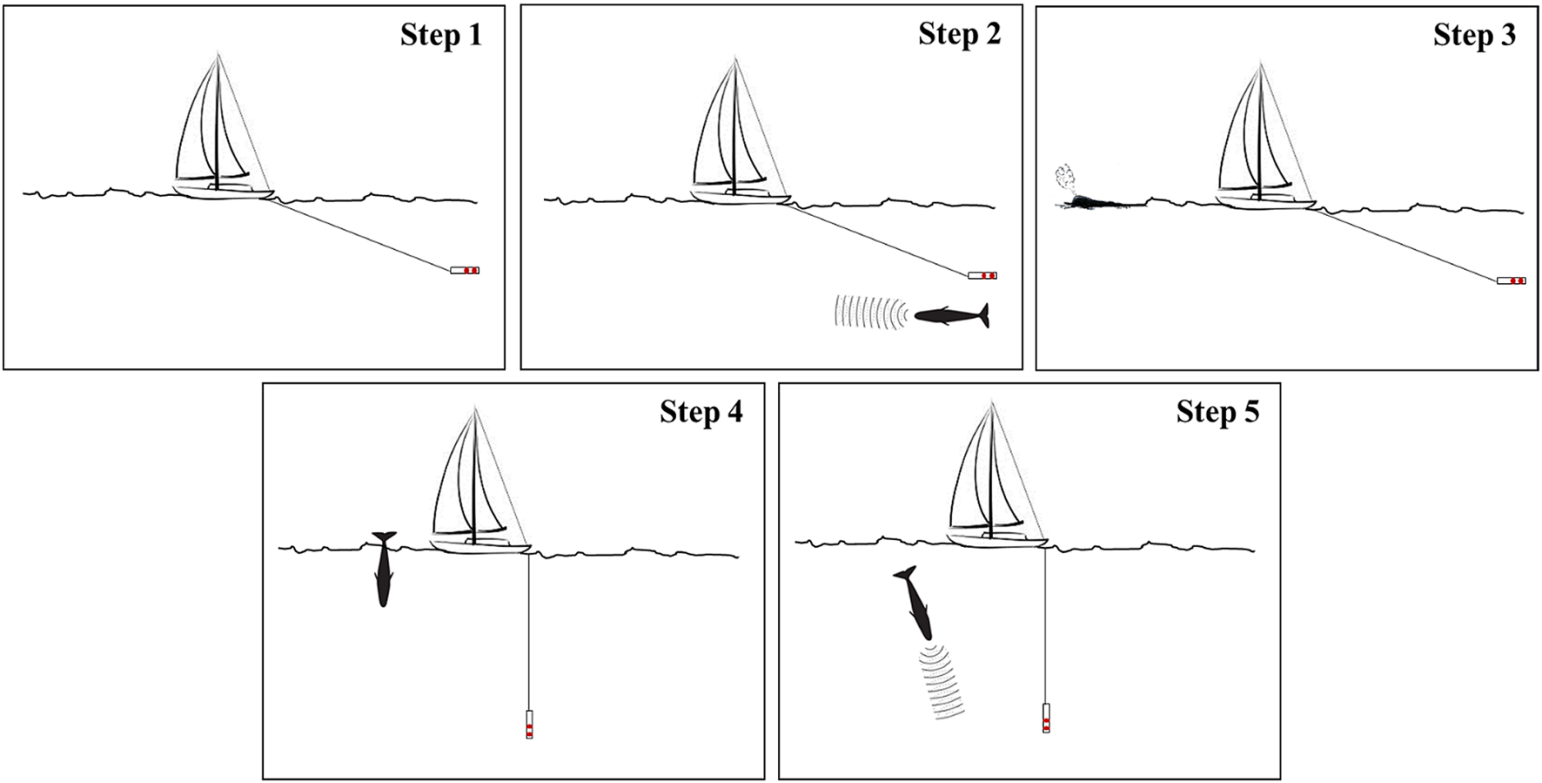
Sequence of activities adopted during the field work to univocally identify each focal whale. Step 1: Whale acoustic detection during surveys; Step 2: Whale acoustic tracking; Step 3: Surface visual detection; Step 4: Photo-identification; Step 5: Recordings.

During the surface period, the focal whale was approached to collect photo-identification data by using a Canon digital camera equipped with image stabilized telephoto zoom lens (70–200 mm F2.8). At the beginning of a new dive after the surface period (i.e., when the whale fluked-up^48^), continuous acoustic recordings were initiated by using Sound Emission Analyzer Pro (SeaPro, developed by CIBRA). Patches, nicks, notches, scars, and other marks on the sperm whale flukes were used to identify individuals^49–52^. Photo-identification pictures were then coupled with recordings from the data logging system, in order to associate in real-time, the photo-identified focal whale to the relative acoustic files. The focal whale started its sounds production just after the fluke-up, when it is still in the first tens or few hundred meters below the surface. Accordingly, the sounds produced by the diving sperm whale have a much higher intensity than any other animal eventually present in the nearby. After 20 min, (while recordings were continuously collected) the boat started again to maneuverer to determine the bearing of the vocalizing focal whale. This “second cycle” had the final goal to confirm the identification of the animal and the correct association between the photo-identification and the recording. The entire process of finding, tracking, visually detecting, photo-identifying and recording the focal whale is summarized in the sequence of the activities shown in Fig. 2. A total of 352 sperm whale encounters were completed by Tethys during the study period (2007–2018), where 149 different individuals were photo-identified.

As for the CIBRA dataset, in September 1996, a 12-day research cruise was conducted in the Pelagos Sanctuary. Due to bad weather conditions, the acoustic survey effort was limited to 7 days. A hydrophone dipole array^53^ was towed with 150 m long cable by a 16 m schooner at a speed of 6 knots. The hydrophones in the array, spaced 8 m to give directional cues, recorded on a DAT recorder (Casio DA-2, 48 kHz sampling rate, 16-bit resolution). Towed array operations totalled 73 h, during which the array was monitored for at least 5 min every 30 min, on a 24-h basis. When sperm whales were detected, continuous monitoring and recording were activated. Sperm whales were then tracked acoustically and eventually approached at surface to obtain photo-identification images (a 35 mm film camera equipped with zoom lens 80–200 mm F2.8 was used) and close-range sound recording. A total of 32 h of DAT recordings were taken in two areas, on the Ligurian coast off Imperia and NW of Corsica off Calvi, where most of the encounters occurred. A group of 3 sperm whales was acoustically detected, tracked and approached off Calvi. Among series of usual clicks and codas, trumpets were also recorded from the same direction of the whales, but individual attribution was not possible.

### Data analysis

A total of 765 h of recordings in 1091 wav files were investigated for trumpets. A total of 230 trumpets (226 TRI; 4 CIBRA) were detected in 227 wav files, through 122 h of recordings. Trumpet data presented here is related to these recordings only, coupled with photo-identification of the corresponding fluking-up (focal) whale whenever possible.

**Figure 3:**
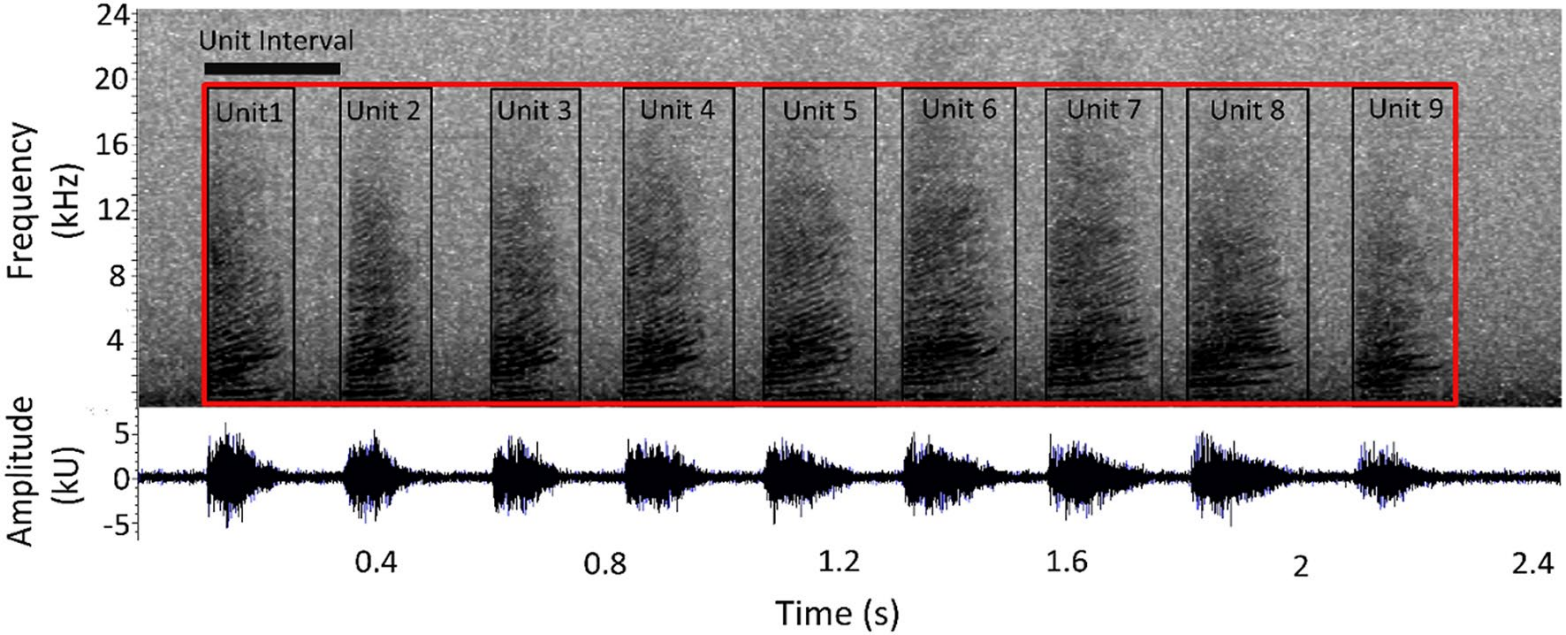
Spectrogram of a sperm whale trumpet using Raven 2.0 (FFT and Hanning window size 2048, 50% overlap).

**Figure 4:**
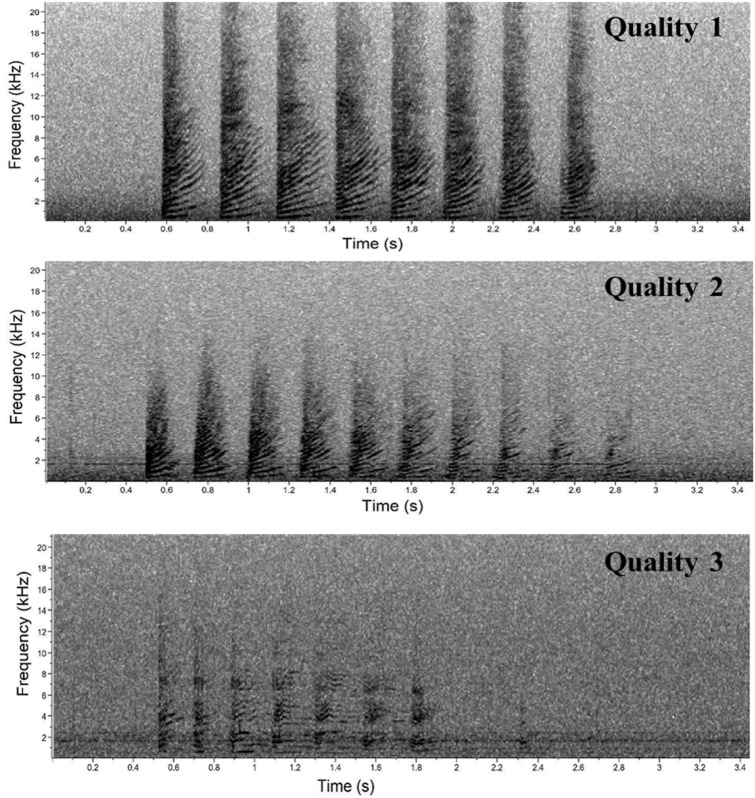
Spectrogram of sperm whale trumpets scored as quality 1 (upper panel), 2 (middle panel) and 3 (lower panel) using Raven 2.0. Upper and middle panels (FFT and Hanning window size 1024, 50% overlap), lower panel (FFT and Hanning window size 512, 50% overlap).

Trumpets resulted in sound elements composed by a rapid series of up-sweep units with extended harmonic structure with no apparent formants (Fig. 3). Different acoustic parameters (Table 2) were measured for each Trumpet and for each Unit in a Trumpet using Raven Pro Sound Analysis Software^54^. Depending on the acoustic parameters successfully measured, a quality score of 1 (High), 2 (Medium) or 3 (Low) was assigned to each trumpet (Table 2 and Fig. 4).

**Table 2:** Acoustic parameters measured for each trumpet and for each unit in a trumpet. A quality score from 1 (high) to 3 (low) was assigned to each trumpet depending on the acoustic parameters successfully measured (X). Raven 2.0 was used for the analysis.

Parameter	Description	Quality 1 (high)	Quality 2 (medium)	Quality 3 (low)
Trumpet initial fundamental frequency (Hz)	Fundamental initial frequency of the first unit of each trumpet	X	X	X
Trumpet final fundamental frequency (Hz)	Fundamental final frequency of the last unit of each trumpet	X		
Trumpet duration (s)	Manual measure of the total duration of each trumpet	X	X	
Number of units per trumpet	Number of single detectable units composing each trumpet	X	X	X
Unit interval (s)	The time interval between the beginning of a unit and the beginning of the immediately following one	X	X	X
Unit repetition rate (N s^−1^)	1/unit interval	X	X	
Unit duration (s)	Manual measure of the duration of each unit in a trumpet	X	X	
Unit duration 90% (s)	Unit duration based on 90% of the signal energy	X	X	X
Unit initial fundamental frequency (Hz)	Initial fundamental frequency of each unit composing the trumpet	X	X	
Unit final fundamental frequency (Hz)	Final fundamental frequency of each unit composing the trumpet	X

Spectrograms were generated with different settings depending on the sample rate of the recordings and the analysis. For all recordings collected at 44.1 kHz, FFT and Hanning window size of 512 was used to measure the Trumpet parameters, and FFT and Hanning window size of 1024 to measure the Unit parameters. For all recordings collected at 96 kHz, FFT and Hanning window size of 2048 used to measure the Trumpet parameters, and FFT and Hanning window size of 4096 for measuring the Unit parameters. Overlap of 50% was chosen for all different settings. A measurement rectangle was manually traced around each Trumpet, and for each Unit in the Trumpet, to assess acoustic parameters (Fig. 2). Duration-90% was introduced in the Unit analysis. This parameter was automatically computed as the time interval containing 90% of the signal energy (i.e. the difference between time points marking 95% and 5% of spectrogram power spectral density) in the rectangle selection drawn in Raven and was introduced to limit the variability and the potential errors that could be generated by manually identify the points on the spectrogram^55,56^.

The unit interval was calculated as the interval between the onset of two consecutive units^57^. The Unit Repetition Rate in a trumpet, in unit per second, was computed as 1/unit interval. Duration parameters were automatically extracted by the software while frequency parameters were manually measured moving the cursor on the spectrogram and selecting the most reliable measure points. The initial fundamental frequency and the final fundamental frequency were measured on the fundamental frequency when visible as the starting and the ending point of the Units composing the Trumpet, otherwise these parameters were estimated by measuring the frequency interval among visible harmonics.

**Figure 5:**
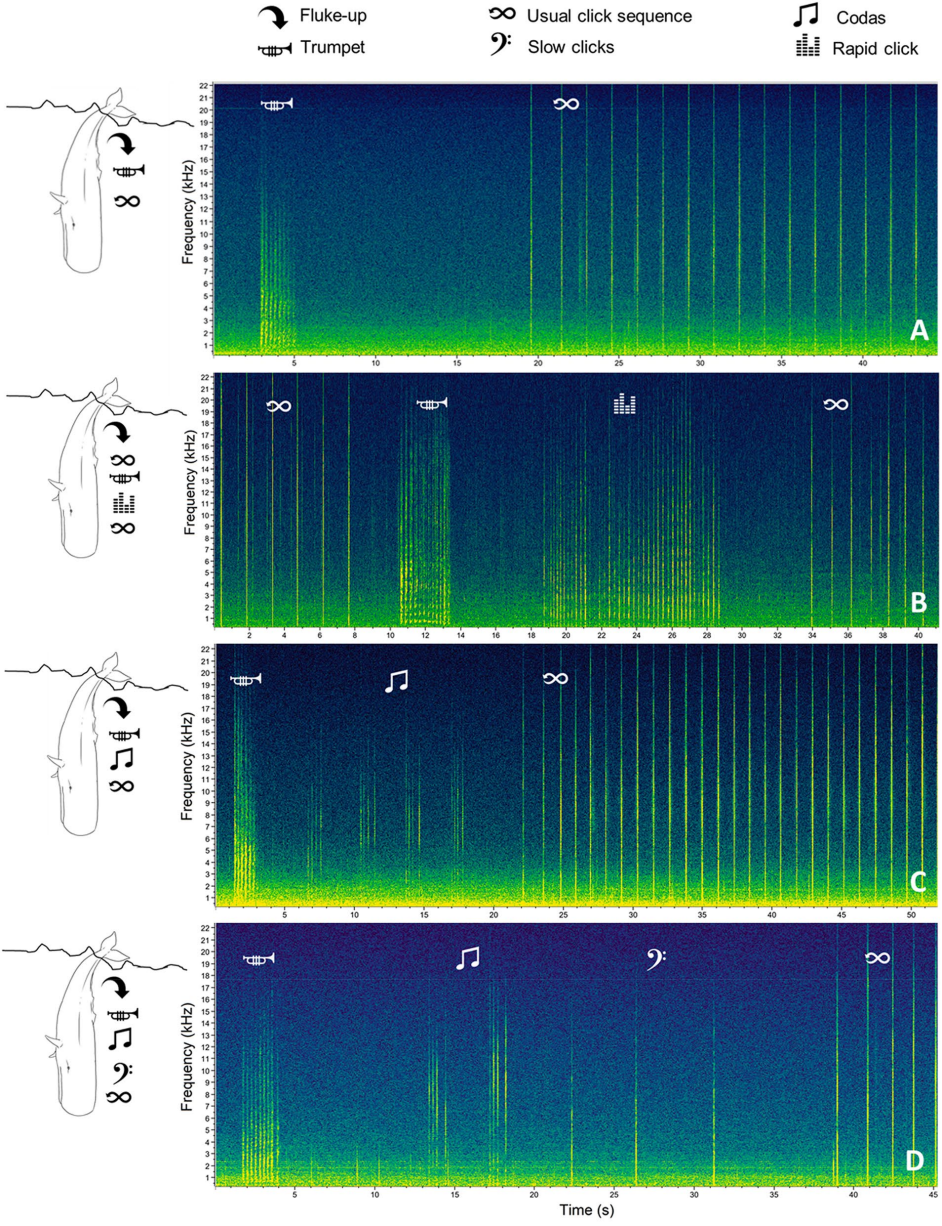
Spectrograms showing the Acoustic Events associated with trumpet emissions using Raven 2.0 (FFT and Hanning window size 1024 (**A**) and 512 (**B**, **C**, **D**), 50% overlap). **A** Regular; **B**, **C**, **D** multi-pattern arrangements.

Details on the context during trumpet recordings were also collected and reported as: DT interval (the time in seconds from the focal whale fluke-up to the onset of the trumpet), TFC interval (the distance in seconds from the end of the trumpet to the first usual click emitted by the focal whale), the estimated Group Size scored as 1 (the focal whale only) or > 1 (other whale(s) than the focal one visually/acoustically detected during each recording containing a trumpet), and the Acoustic Events (the of presence of other sounds around the trumpet after the focal whale fluke-up and before the start of the usual click sequence). The Acoustic Events were defined as “Regular” (a trumpet followed by an acoustic pause and then a series of usual click) and “Multi-Pattern” (a trumpet preceded/followed by different kind of click patterns, such as short sequences of 1–8 slow clicks with an inter-click-interval ≥ 2 s, codas, and/or rapid clicks; Fig. 5). For a subset of 214 trumpets (hereafter referred to as “Subset”), it was possible to assess both the Acoustic Events and the identity of the focal whales emitting trumpets (“Trumpet Whales”; n = 68) through photo-identification. The Acoustic Events in the 68 Trumpet Whales were assessed in both recordings with and without trumpets.

Maps were generated using the software QGIS (Version 2.18.16). Slope values were calculated using the ESRI Arcview Spatial Analyst tool^58^ and depth data was derived through the GEBCO One-minute Digital Atlas (https://www.gebco.net/data_and_products/gridded_bathymetry_data/gebco_one_minute_grid/).

### Statistical procedures

Given the multilevel structure of the data (Units composing a Trumpet, and Trumpets nested in Individuals), a Linear Mixed Model (LMM) approach^59^ was applied to test the variation of the acoustic parameters (Table 3) within the same individuals and or between different individuals. Only high-quality Trumpets from 14 Trumpet Whales were used, choosing as the random effect the Trumpets nested in the Individuals (Model 1), Individuals only (Model 2), and Trumpets only (Model 3); the Initial Fundamental Frequency of each Unit was selected as the fixed effect (independent variable) and the Duration of each Unit was used as the dependent variable.

Overall, variations in the Trumpet acoustic parameters were examined in relation to Group Size (2 classes: 1 or > 1 whale) and Acoustic Events (2 classes: Regular or Multi-Pattern) using two-tailed Welch’s t-tests. The influence of Environmental Variables (2 classes: Depth and Slope) on Acoustic Events was examined through paired t-test. A binomial logistic regression approach^60,61^ was employed to model the presence/absence of the Trumpet by using the Group Size and the Acoustic Events as predictors. A Pearson's chi-squared test was then applied to analyse the relationship between the Trumpet occurrence and the different click patterns (codas, rapid clicks, and slow clicks) in the Acoustic Events.

Analysis was performed in R (version 3.3.3, The R Foundation for Statistical Computing, Vienna, Austria, http://www.r-project.org) using CRAN packages Seewave 2.1.5^62^, ggplot2^63^, dplyr 0.8.5^64^, lme4^65^, lmerTest^66^, and SPSS Statistics (version 26, IBM, New York, USA, https://www.ibm.com/it-it/products/spss-statistics).

## Results

### Overall

Trumpet recording locations are reported in Fig. 1. All trumpets (n = 230) were recorded at the beginning of a new dive after the focal whale fluked-up following a period at the surface. The time interval from the beginning of a dive (after the fluke-up) and the trumpet (DT interval) had an average of 35.5 s, ranging from 1.8 to 131.5 s. The time interval from the end of the trumpet to the onset of the first usual click (TFC interval) averaged 18.22 s, ranging from 2.6 to 77.7 s.

Over a total of 230 analysed trumpets, 44 were scored as high-, 112 as medium- and 74 as low- acoustic quality. The initial frequency of each trumpet, measured on the first unit, ranged from 245 to 649 Hz, whereas the final frequency, measured on the last unit, ranged between 301 and 964 Hz (Table 3). As expected, the total duration of the trumpet was strongly related to the number of units, lasting between 1.1 and 6.6 s, per number of units ranging from 2 to 24 (Table 3). The Unit Repetition Rate in a trumpet showed an average of 3.9 s^−1^, ranging from 1.4 to 5.9 s^−1^. Trumpet durations were significantly longer in the Multi-Pattern (mean = 2.63 s) than the Regular Acoustic Events (mean = 2.36 s) (Welch’s t-test: *t* (213) = 2.8838; *p* < 0.01; Supplementary Figure S1). About 89% of the trumpets (n = 204) were documented when whale(s) other than the focal one was visually/acoustically detected during each recording with a trumpet (Group Size > 1). This proportion is comparable with recordings without trumpets. The trumpet initial frequency was significantly higher when Group Size was > 1 (mean = 439 Hz) than when there was just one whale in the area (mean = 412 Hz) (Welch’s t-tests: *t* (124) =  − 2.1704), *p* < 0.05; Supplementary Figure S2).

The Units composing the trumpet were characterized by increasing frequency (sweep-up) throughout their duration. Their initial frequency ranged from 212 up to 672 Hz, whereas the final one between 332 and 1774 Hz (Table 3), regularly increased throughout each unit. Units lasted an average of 0.21 s, ranging between 0.11 and 0.52 s. The average Unit Interval was 0.26 s, ranging between 0.15 up to 0.61 s.

**Table 3:** Descriptive statistics of the trumpets’ acoustic parameters.

Parameter	Min	Max	Mean ± SD	Confidence interval 95%
Trumpet initial fundamental frequency (Hz)	245	649	430 ± 72	439–416
Trumpet final fundamental frequency (Hz)	301	964	625 ± 162	733–511
Trumpet duration (s)	1.1	6.6	2.5 ± 0.8	2.7–2.4
Number of units per trumpet	2	24	10 ± 3	10.4–9.6
Unit interval (s)	0.15	0.61	0.26 ± 0.08	0.27–0.26
Unit repetition rate (N s^−1^)	1.4	5.9	3.9 ± 0.6	3.9–3.8
Unit duration (s)	0.11	0.52	0.21 ± 0.05	0.21–0.20
Unit duration 90% (s)	0.02	0.27	0.13 ± 0.04	0.14–0.13
Unit initial fundamental frequency (Hz)	213	672	388 ± 66	392–384
Unit final fundamental frequency (Hz)	332	1764	795 ± 219	819–771

**Figure 6:**
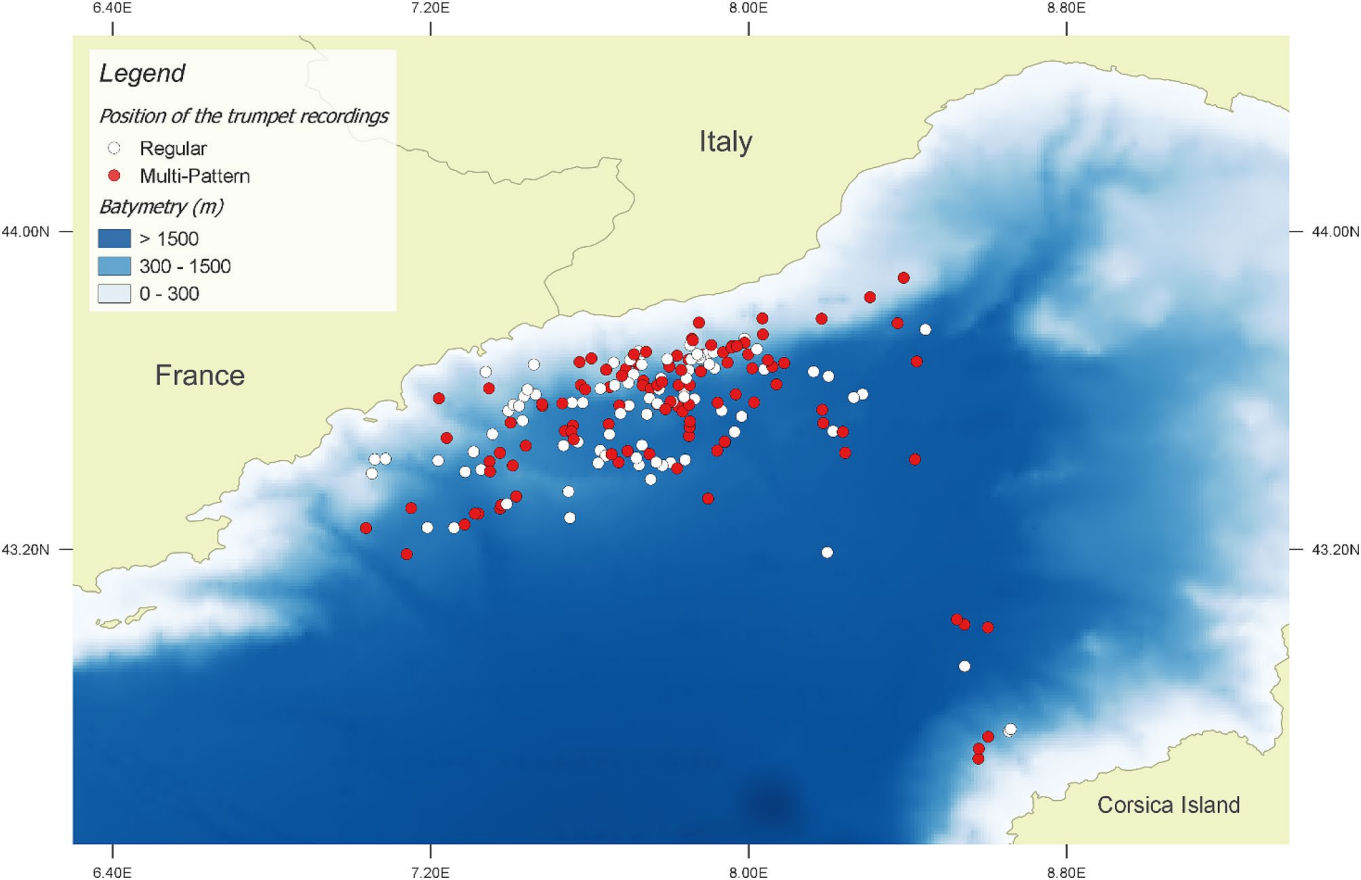
Position of the sperm whale TRI trumpet recordings showing Regular (white dots) or Multi-Pattern (red dots) Acoustic Events. Map created using the Free and Open Source QGIS.

Unit duration 90% resulted higher in the Multi-Pattern than in the Regular Acoustic Events (Pair t-test: *t* (213) =  − 16.339; *p* < 0.001). The Acoustic Events were mapped (Fig. 6) and related to Environmental Variables (i.e. depth and slope descriptive statistics). Multi-Pattern series turned out to be recorded in areas with higher slope values (Pair t-test: *t* (213) =  − 13.521; *p* < 0.001) and lower slope variability (Pair t-test: *t* (213) = 14.083; *p* < 0.001), i.e. submarine canyons (Supplementary Figure S3).

### Subset

As previously mentioned, for a subset of 214 trumpets, 68 Trumpet Whales were identified over a total of 149 that were catalogued in the study period. The proportion of the Trumpet Whales represented more than 50%, and up to 75% of the total number of photo-identified whales each year (Supplementary Table S1). The individual emission rate (Table 4)—calculated as the number of encounters with trumpets over the total number of encounters per focal Trumpet Whale—has an average of 0.58. For almost 62% (n = 42) of the Trumpet Whales, more than one trumpet in the same survey season or in different ones was recorded, with 29 individuals producing trumpets over different years (maximum range: 10 years).

**Table 4:** Summary of the trumpet characteristics in the Trumpet Whales.

#	Trumpeting Whale	N. of recorded trumpet	Trumpet years	Trumpet rate	Trumpet initial fundamental frequency (Hz)	N. of units in a Trumpet	Trumpet duration (s)	Unit repetition rate (N s^−1^)
1	ALBERT	2	2009, 2011	0.40	396	7.5	1.87	4.03
2	AMER	1	2018	1.00	389	7.0	1.97	3.56
3	ANDERSON	1	2007	1.00	402	10.0	2.60	3.85
4	BIOLU	2	2013, 2017	0.40	649	10.0	2.57	3.91
5	BIOS	1	2007	0.14	461	8.0	1.85	4.32
6	BLINKY	2	2012	0.33	NA	10.5	2.61	3.76
7	BRIC	5	2011	0.27	NA	5.5	1.32	NA
8	CABEZON	2	2008	1.00	515	10.0	2.61	3.81
9	CHARLES	2	2011	0.50	NA	8.0	2.06	3.65
10	CHILI	2	2012, 2013	0.40	509	10.5	2.68	4.15
11	COM	3	2012	0.50	371	9.3	2.38	3.61
12	CONCA	1	2012	0.50	NA	7.0	1.66	NA
13	DARENOME	3	2016	1.00	374	7.7	2.07	3.61
14	DAVE	8	2013, 2014	0.50	388	10.5	2.83	3.66
15	DEVIL	1	2012	1.00	NA	9.0	1.98	NA
16	ENRICO	6	2008, 2011, 2015	0.33	387	10.3	2.66	3.97
17	FEDE	1	2011	0.33	NA	12.0	3.13	NA
18	FLO	1	2009	0.50	328	7.0	1.63	4.07
19	FOCONE	5	2010, 2011	1.00	385	14.2	3.66	3.77
20	FOURTY	4	2008, 2009	0.50	458	11.8	2.89	4.02
21	FRANCI	1	2011	0.25	511	10.0	2.41	NA
22	FREDDY	3	2009, 2012	0.29	501	9.3	2.59	3.48
23	GABI	1	2010	0.25	433	6.0	1.59	NA
24	GEORGE	1	2010	1.00	NA	8.0	1.87	4.30
25	GINKO	1	2009	1.00	433	8.0	2.05	NA
26	GOBBA	1	2012	0.25	NA	6.0	1.72	3.73
27	GOGO	7	2008, 2011, 2016, 2017	0.38	450	9.4	2.41	NA
28	HAL	1	2013	0.50	514	7.0	3.92	NA
29	HALF	2	2009	1.00	494	12.5	3.42	3.45
30	HARALD	6	2009, 2013, 2014, 2015, 2017	0.30	413	10.2	2.63	3.89
31	HENRY	6	2010, 2011, 2015	0.57	385	8.5	1.92	4.43
32	HOLGER	1	2011	0.33	554	9.0	2.33	NA
33	IKE	1	2011	0.20	484	11.0	2.88	3.70
34	ISOSCELES	4	2009, 2017	0.50	368	10.3	2.57	3.80
35	JOHN	1	2010	1.00	NA	11.0	2.58	4.15
36	KIKI	1	2016	1.00	503	9.0	2.06	4.45
37	LEIRE	2	2007	0.50	377	10.0	2.41	3.93
38	LINUS	1	2009	0.50	528	11.0	2.68	4.05
39	LUCKY	4	2011, 2014	0.67	450	12.0	2.75	4.22
40	LUIGI	1	2013	0.50	406	6.0	1.58	3.91
41	LUKE	1	2009	0.33	NA	8.0	1.89	3.71
42	MARIKO	4	2013, 2014	0.67	473	10.5	2.64	3.67
43	MATT	16	2007, 2008, 2009, 2010, 2011	0.40	379	8.6	2.21	3.85
44	MOUSSE	5	2014, 2017, 2018	0.27	430	10.8	2.80	3.90
45	NEMBO	11	2010, 2011, 2014, 2016	0.64	421	12.2	2.84	4.20
46	NICOLA	11	2008, 2011, 2014, 2015	0.60	396	11.1	2.94	3.77
47	NONI	4	2009, 2010, 2018	1.00	473	10.5	2.68	NA
48	OLIVIER	4	2009	0.60	418	9.5	2.50	3.62
49	OVER	1	2012	0.50	NA	10.0	1.87	NA
50	PAT	2	2009, 2012	0.22	403	11.5	2.84	3.66
51	PELAGOS	2	2007	1.00	454	8.5	2.09	3.41
52	POLLOCK	1	2012	0.50	534	10.0	2.74	3.68
53	PROUST	1	2015	0.25	334	7.0	1.84	4.56
54	RANDY	1	2010	0.25	437	8.0	1.81	3.65
55	REGOLO	3	2014, 2017	1.00	419	8.0	2.03	3.83
56	ROMAN	4	2010, 2017	1.00	385	10.3	2.63	NA
57	ROSS	1	2007	1.00	399	21.0	4.36	3.83
58	SHRECK	4	2015	0.40	522	8.3	1.89	4.01
59	STAMPA	4	2012, 2013	1.00	531	13.3	3.16	4.22
60	STEFANO	2	2007, 2011	0.50	437	11.5	2.58	4.52
61	SYLVAN	2	2013	0.50	455	5.5	1.64	3.72
62	TANTUM	7	2011, 2012, 2016, 2017	0.56	342	10.9	2.63	3.72
63	TIEDIE	1	2012	0.50	NA	14.0	2.94	NA
64	TIM	7	2007, 2010, 2014, 2016	0.63	406	9.6	2.44	3.83
65	TIP	3	2007	0.67	396	9.0	3.20	3.38
66	TOM	5	2007, 2011	0.50	403	8.8	2.13	4.02
67	WALTER	5	2008, 2009	0.60	521	9.2	2.21	4.27
68	ZORO	3	2013, 2016	0.67	488	10.0	2.66	3.97

Three different LMMs were run to test the differences of the acoustic parameters within and between individuals (Table 5). Comparison of the AIC values showed that Model 1 (the one using the Trumpet nested in the Individual as random effect) better explained the correlation between the variables (AIC = 565.70), followed by the Model 2 (ΔAIC = 27.78). The Duration of each Unit significantly correlated with the Initial Fundamental Frequency (t-value = − 3.772; *p* < 0.001) and the variability of the Trumpet nested in the Individuals (SD = 0.67) was higher than between different Individuals (SD = 0.20). The model diagnostics considered the variable independence assessment and the residuals normal distribution (Shapiro–Wilk normality test: W = 0.988; *p* > 0.05).

**Table 5:** Results of the Linear Mixed Model analysis (IFF = initial fundamental frequency; I = individuals; T = trumpets). Model 1 (in bold) is selected as it better explains the correlation between the variables.

Model	Description	AIC	ΔAIC	Intercept (β_0_)	Fixed IFF (β_1_)	σ^2^ (I/T)	σ^2^ (I)	σ^2^ (Residuals)
**1**	**Time = β0 + β1(IFF) + R(I/T)**	**565.70**	**0**	**0.04726**	**− 0.23083**	**0.674**	**0.2032**	**0.7299**
2	Time = β0 + β1(IFF) + R(I)	601.38	27.72					
3	Time = β0 + β1(IFF) + R(T)	629.10	36.60					

The AIC value is also reported for Model 2 and 3.

The stepwise binary logistic analysis selected the Acoustic Events as the strongest predictors of Trumpet occurrence (Table 6). As shown in the confusion matrix, the model had a higher accuracy for predicting Trumpet presence (95%) and a lower accuracy for Trumpet absence (52%). However, the overall accuracy of the model was higher than 75% (Table 6b). Regular Acoustic Event inversely correlated with the Trumpet presence (Table 6a), suggesting that the Multi-Pattern Acoustic Events could be more associated with the trumpet emission than the Regular. Finally, Pearson chi-squared test highlighted a significant association between trumpet occurrence and sequences of slow clicks in the Multi-Pattern Acoustic Events (χ^2^ (1, 505) = 148.9, *p* < 0.0001).

**Table 6:** Results of the binary logistic regression model: (a) presence/absence of Trumpet were correlated with the occurrence of the Acoustic Events (Regular and Multipattern); (b) confusion matrix showing the logistic models classification performances of Trumpet presence/absence; the overall percentage of presence/absence classification is shown in bold.

(a) Binary logistic regression parameters					
	B	S.E	Wald	df	*p*
Regular	− 3.074	0.306	100.597	1	0.000
Costant	2.079	0.283	53.811	1	0.000

(b) Confusion matrix					
Observed	Trumpet absence	Trumpet presence	Overall %		
Trumpet absence	273	14	95.1		
Trumpet presence	101	112	52.6		
Overall %			**77**		

The cut value is 0.5.

## Discussion

Here we explored a topic that was scarcely reported until now. Specifically, we investigated the sperm whale trumpets in the Mediterranean Sea, their acoustic characteristics, the context of emission, and the individual variability of these sounds.

Acoustic emissions generated by specialized anatomical structures are often presumed to be signals, even if their functional purpose is unclear or undetermined^25^. A signal evolved to deliver information that, on average, enhances long-term fitness of both the signaller and receiver(s)^67–69^. The aim of a signal (or of a signaling system) is communication^70^, and its goal is to change the receiver’s behavioural, physiological, or developmental responses^71^. If any information is obtained from traits that are not signals (i.e. not evolved for the purpose of conveying information), these traits are reported as cues^25,72^.

It is not easy to demonstrate if a sound is a putative signal or a cue, as different acoustic cues can be a source of information beneficial to both sender and receiver as well, and some vocal signals may have evolved from altered breathing patterns that once were cues^24,68,69,73^. It is still unclear if sperm whale trumpets are sound signals conveying informative aspects of the signaller (e.g., individual/species/population identity, age, physiological condition or motivation^73^) or the context (referential signaling^73^), or if they are cues, by-products of the click generation mechanism^9,18^. While the precise trumpet producing mechanism is still unknown, the by-product hypotheses may have been inferred from the extended harmonic series in the trumpets, which suggest an underlying pulsed structure possibly related to the click production. The suggestion that the trumpet could be produced by a threatened whale as an alarm call due to the presence of the vessels^9,16^ introduce the signal hypotheses. This may derive from the well recognizable and stereotyped acoustic structure of the trumpet. Stereotypy is defined as one of the necessary components of signal evolution, to increase consistency of signal perception for effective communication and to allow receivers to reliably associate a particular signal with a conspecific^66^, or with a particular individual (i.e.*,* the vocalization that contains sufficient unique information to be individually distinctive and with a specific situation^74^). Consequently, stereotyped sounds are commonly used as a way of individual recognition in a wide range of taxa, including mammals (e.g., Australian sea lion*, Neophoca cinerea*)^75^; African elephant, *Loxodonta africana*^76^). From the existing data, however, it is difficult to understand which, if any, of the signal or cue hypotheses reflects the function of trumpets.

### Hint #1: Trumpet stability

The results of this study indicated that trumpets in the Mediterranean are conserved in the sperm whale acoustic repertoire at the decadal timescale, demonstrating the persistence of these sounds over 22 years (1996–2018). Presumably, if a sound has a relevant function in a given context, it would be disadvantageous to change it over time. Even so, trumpets were not recorded for all individuals in all encounters during each dive, nor were they recorded in all dives by the same whale. The absence of trumpets, however, did not necessarily imply that they were absent from the individual repertoire, since it is unlikely that the recordings collected in this study comprise the whole acoustic repertoire of all whales of the entire population at any given time. Our results showed that at least half of the known whales frequenting the study area emitted trumpets each year, indicating the persistence of this sound type in space and in the same individuals across a wide time period. This stability was reported by Pace as well^19^ in the Tyrrhenian Sea (Italy), advancing the hypothesis that trumpets may be a long-lasting component of the individual acoustic repertoire.

### Hint #2: Trumpet variability in social context and different individuals

This study reported the persistence of the trumpets in individuals over time, but a variability in their fine-scale structural parameters with the context. The Initial Fundamental Frequency of the units and of the entire trumpet was significantly higher when more than one whale was visually/acoustically recorded (Group Size > 1) during the trumpet emissions. Higher fundamental frequencies seem to be mainly related to intense social interactions^77,78^. The African elephant, for example, produce sounds at a higher fundamental frequency during positive social situations and dominance circumstances^79^. The increase of the initial fundamental frequency here reported in social context might reflect a situation of “excitement” experienced by the whales when other individuals were detected in the area, a hypothesis that is further supported by the correlation between the initial fundamental frequency and the duration of the units in 14 Trumpet Whales. In intense social contexts, other species like baboons (*Papio* sp.)^80^ and chimpanzees (*Pan troglodytes*)^81^ are reported to produce specific sounds both longer and at higher fundamental frequencies than the ones emitted in more relaxed situations. The higher initial fundamental frequency and the duration variability within each Trumpet Whales further suggested an individual plasticity in composing and arranging units in a trumpet.

### Hint #3: Trumpet association with social click patterns

The results of this study showed that the trumpet emission was related to different sequences of click patterns. Trumpets were reported to be produced at the beginning of a dive, prior to the usual click series onset^12,15–18^. This was true for most of the trumpets analysed in this study although, on eleven occasions, the sequence of usual clicks began before the trumpet’s emission. In our knowledge to date, this is the first time that an observation of this kind has been reported. Teloni^17^ illustrated that in some instances the trumpet was preceded by codas, as also observed in this study in one case, and that a few trumpets were followed by a short sequence of clicks (usually 2) with an inter click interval of about 5 s. No mention in the literature was found of codas or other social vocalizations, such as rapid clicks, following the emission of the trumpet, as here reported. It was postulated that the sperm whale can change the acoustic characteristics of the sound generated in the nasal complex when switching between codas and echolocation clicks, two highly different click patterns in terms of directionality and acoustic output^9^. Based on the description of their acoustic structure and considering that they are emitted at the beginning of a deep dive, Gordon^9^ hypothesized that trumpets may be physiological sounds generated by the complex respiratory system of the whale in preparation for immersion. Teloni and colleagues^18^ further proposed that trumpets might be a by-product of airflow in the vocal tract of the sperm whale as it modifies the sound production apparatus from a configuration appropriate for respiration and surface codas, to one appropriate for diving and sonar clicks. Observations from this study, which identified Regular and Multi-Pattern Acoustic Events (i.e. sound sequences around trumpet including social sounds such as codas and slow clicks), provided evidence of a more complex scheme than previously described, with trumpets being emitted both before and after the onset of either codas (and other social sounds) or usual echolocation clicks. In the majority of the Multi-Pattern Acoustic Events here reported, the trumpet was followed by some codas and, more frequently, by short sequences of 1–8 slow clicks^4,11,12^, a click type—only recorded in males—that has a possible long-range communicative function (e.g., presence, location, identity and perhaps size of the signaller^8^) due to both the long inter-click interval and the waveform^11^. In addition, the trumpet duration (and so the number of units) and Units Repetition Rate were longer in the Multi-Pattern Acoustic Events. More stereotyped repeated units mean greater redundancy, and greater redundancy may improve sound detectability^82^.

### Hint 4#: Trumpet association with steep slope habitat and foraging dives

Trumpets may also persist in the sperm whale repertoire in the North-western Mediterranean because they may be functionally specific to foraging activities in this region. It is widely recognised that sperm whales travel through the whole western Mediterranean Sea, with movements and exchanges of males within the area^50,83^. Male sperm whales use the North-Western Mediterranean Sea especially in the summer months, while social units of females with calves and juveniles tend to remain in southern areas^84–86^. Foraging appears to be the predominant activity performed by sub-adult/adult males while they are at the higher latitudes^32,33,87,88^, with a strong relation with the continental slope area^30,40,41,89^. In particular, sperm whale habitat preference seems associated with submarine canyons^32,44,87^, known to be extremely productive, comprising complex topographic features which serve as hotspots of biodiversity and key habitats for top predators^90^. The trumpet datasets analysed here were collected in a high-latitude habitat that is used predominantly as foraging ground for sperm whales, with higher foraging rates occurring in steep slope habitat^87^ (i.e. a proxy of canyon systems). The Multi-Pattern Acoustic Events, with codas and slow clicks associated with the trumpet, appear to be produced in areas where the slope is steepest, suggesting a potential occurrence of communication dynamics among individuals at the beginning of a foraging dive. Coupling the linear distance between two different sperm whales emitting trumpets during the same survey (6.6 km; Supplementary Table S2), and the frequent association of the trumpet with slow clicks that potentially relays information regarding individual identity or behavioural states^12^, may be indicative of a long-range communication between males in a Mediterranean high latitude feeding ground.

## Wrap-up

This study provides the first evidence that sperm whale trumpets may persist across years and individuals in the same area. The higher fundamental frequency when multiple whales were visually/acoustically detected, the stereotyped characteristics of the trumpet acoustic structures, the frequent association with a male only communication signals (i.e., slow clicks), and the emission in feeding grounds, suggest the trumpet functions as a sound of maturing/mature males, perhaps having a role in male-male interaction context during foraging. Even with these totally new findings, it is still not possible to assess which one contributed most to the hypotheses of trumpet as a signal (intentional signal either to label an individual or a situation) or a cue (unintentional conveyers of information). In the absence of a more consistent dataset this remains an open question. Further investigation is needed and is strongly encouraged to better understand the role of temporally stable trumpets as well as individual variation, and trumpet usage across sex, age and size, social context, noise conditions, and sperm whale populations.

## Supplementary Information

Supplementary Informations.
