# Supplementary material for: Trumpet sounds emitted by male sperm whales in the Mediterranean Sea
Source: Sci Rep. 2021 Mar 12;11:5867. doi: 10.1038/s41598-021-84126-8 (PMC7955081; doi:10.1038/s41598-021-84126-8)
Supplement: Supplementary file 1 — Supplementary Informations. [file 41598_2021_84126_MOESM1_ESM.docx]

**SUPPLEMENTARY INFORMATION**

**Trumpet sounds emitted by male sperm whales in the Mediterranean Sea**

Pace D.S.^1*^, Lanfredi C.^2*^, Airoldi S.^2^, Giacomini G.^1^, Silvestri M.^1^, Pavan G.^3^ and Ardizzone D.^1^

^1^ Department of Environmental Biology, Sapienza University of Rome, Italy

^2^ Tethys Research Institute, Italy

^3^ Department of Earth and Environmental Sciences, CIBRA, University of Pavia, Italy

* Corresponding authors

^1^Daniela Silvia Pace

[danielasilvia.pace@uniroma1.it](mailto:danielasilvia.pace@uniroma1.it)

^2^Caterina Lanfredi

lanfredicaterina@gmail.com


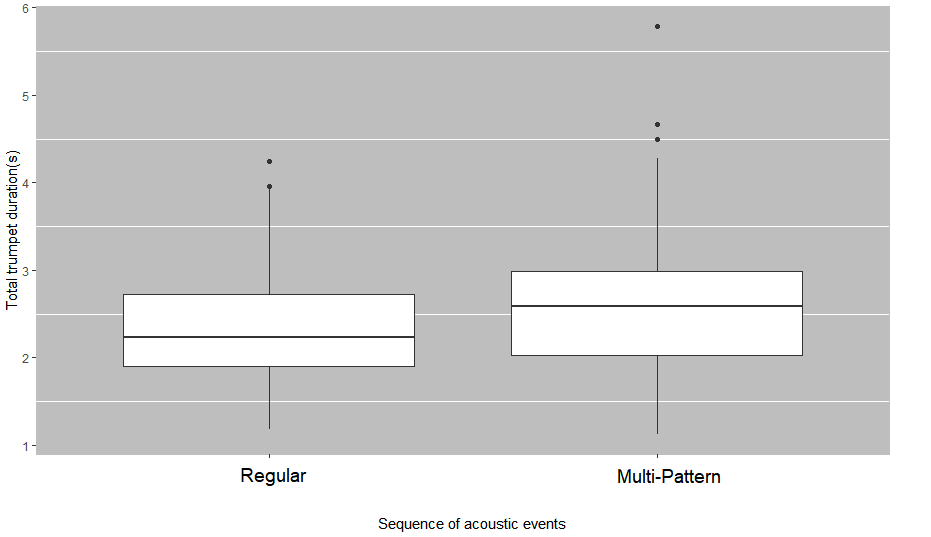


Figure S1. Trumpet total duration in relation to the Acoustic Events (Regular and Multi-Pattern).


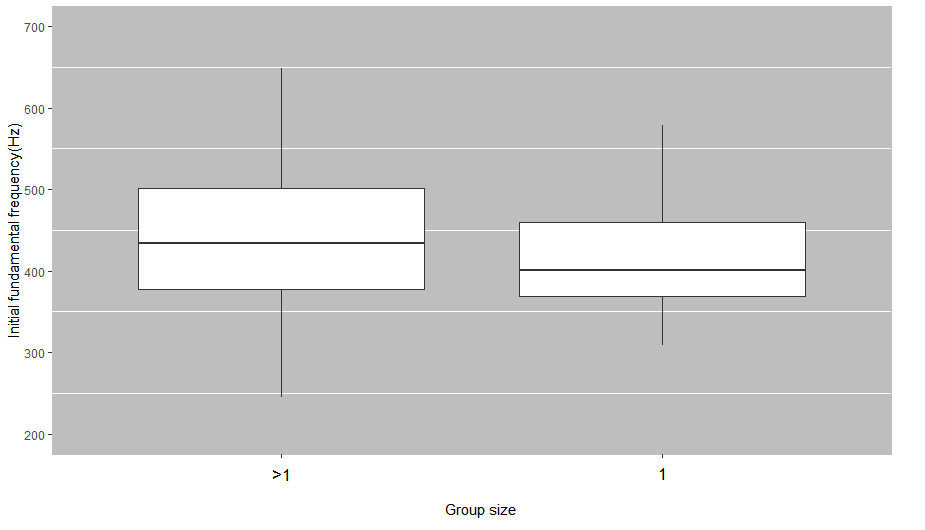


Figure S2. Trumpet initial frequency in relation to Group Size

(categorized as 1 or more than one (>1) individulas).


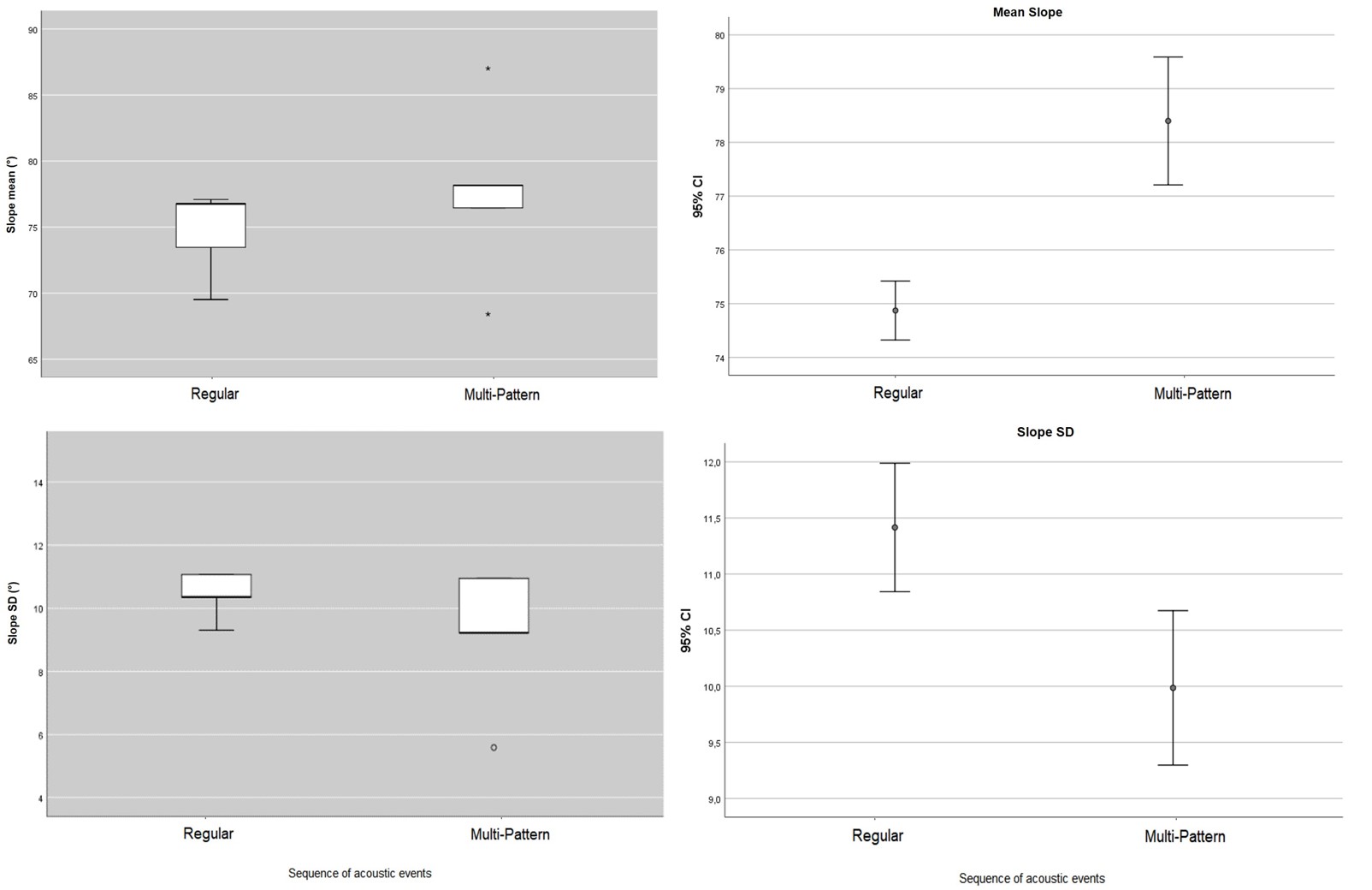


Figure S3. Physiographical features Slope Mean and Standard Deviations (SD) in relation with to the Regular and Multi-Pattern sequences of Acoustic Events.

Table S1. Proportion of the Trumpet Whales over the total number of photo-identified (PhId) whales each year

| **Year** | **N. of PhId whales** | **% of Trumpet whales** |
| --- | --- | --- |
| 2007 | 27 (19) | 70% |
| 2008 | 26 (13) | 50% |
| 2009 | 30 (20) | 67% |
| 2010 | 25 (19) | 76% |
| 2011 | 39 (28) | 72% |
| 2012 | 32 (24) | 75% |
| 2013 | 19 (14) | 74% |
| 2014 | 19 (14) | 74% |
| 2015 | 21 (9) | 43% |
| 2016 | 18 (9) | 50% |
| 2017 | 20 (12) | 60% |
| 2018 | 9 (5) | 55% |

Table S2. Linear distance (km) between different individuals emitting trumpet in the same encounter (n=19 pairs of Trumpet Whales), calculated by using QGIS tool.

|  | **N** | **Min** | **Max** | **Average** | **SD** |
| --- | --- | --- | --- | --- | --- |
| **Linear distance between different Trumpet Whales (km)** | 19 | 1.8 | 21 | 6.63 | 4.7 |
